# Biochemical and Ultrastructural Cardiac Changes Induced by High-Fat Diet in Female and Male Prepubertal Rabbits

**DOI:** 10.1155/2018/6430696

**Published:** 2018-04-04

**Authors:** Dina Sibouakaz, Khira Othmani-Mecif, Amirouche Fernane, Abdennour Taghlit, Yasmina Benazzoug

**Affiliations:** Laboratory of Cellular and Molecular Biology, Team of ECM Remodeling Biochemistry, Faculty of Biological Sciences, University of Sciences and Technology Houari Boumediene, BP 32, EL Alia, 16111 Algiers, Algeria

## Abstract

Early weight gain induced by high-fat diet has been identified as a predictor for cardiac disease, one of the most serious public health problems. Our goal is to study the influence of a HFD on biochemical, oxidant stress parameters, and the cardiac ultrastructure in both male and female prepubertal models. Experiments were carried on 24 prepubertal New Zealand white rabbits, randomly assigned to male and female control (MC and FC, resp.) or HFD (MHFD and FHFD, resp.) groups (*n* = 6) for 3 months. Body and heart weights and some biochemical and oxidative stress parameters such as lipids, calcium, CKMB, MDA, uric acid, ascorbic acid, and AOA are evaluated in plasma and the left ventricle. Under HFD effect, plasma parameters, such as lipids (TL, PL, and LDL-C), MDA, and CK-MB, increase more significantly in male than in female groups, when AA decreases. Some cardiac parameters such as TG and UA increase, when AA and AOA decrease; these variations are more significant in FHFD. In both male and female rabbits, HFD caused changes in heart ultrastructure, junctional complexes, mitochondria size and form, and so on. Early HFD feeding induced overweight, oxidative stress, and metabolic alterations in plasma and the heart of prepubertal rabbits, whereas lipotoxicity has especially a negative impact on male plasma but affects more the female heart ultrastructure.

## 1. Introduction

Early obesity is increasing worldwide and represents a serious health issue with an elevated risk of cardiovascular disease [1, 2]. Numerous clinical and epidemiological studies point to a strong link between body weight and heart disease in several populations. High-fat diets have been correlated with excess body weight and a higher occurrence of hypertrophic cardiomyopathy [3]. However, the influence of body weight on serum lipids is often overlooked in clinical practice. Excess body weight is indeed associated with higher plasma triglyceride, LDL cholesterol, fasting glucose levels, and lower HDL cholesterol content [4, 5]. Moor de Burgos et al. [6] noted a significant decrease in serum antioxidant vitamin levels (ascorbic acid) and a higher prevalence of vitamin deficiency and dyslipidemia in obese women. For Galili et al. [7], early phases of abdominal overweight/obesity are characterized by coronary endothelial dysfunction, vascular oxidative stress, and light lipid profile abnormalities.

Oxidative stress has also shown to play an important role in cardiovascular diseases [8] in association with dyslipidemia and hyperglycemia [9], and it is, in association with metabolic stress, a key factor in the pathogenesis of overweight-related diseases [10, 11]. The cardiac tissue is sensitive to oxidative damage because of its high oxidative capacity and its poor antioxidant enzyme content [12]. The loss of mitochondrial integrity due to oxidative damage eventually disturbs cell functions, sensitizes cells to stress, and triggers cell death with potentially irreversible pathological consequences. A line of evidence suggests that overweight-/obesity-induced cardiac dysfunction is linked to excessive mitochondrial ROS production, oxidative stress, and massive loss of cardiac cells [13, 14].

The beginning of cardiovascular pathology in young individuals is now recognized as a clinical problem [15], and early biochemical determinations prevent numerous cardiovascular diseases [15, 16]. A worldwide increase in the prevalence of juvenile overweight and obesity is currently observed [17], in association with vascular dysfunction in otherwise healthy young children [18]. For Galili et al. [7], the initial cardiovascular manifestations of obesity, including endothelial dysfunction, might start early, before the metabolic syndrome establishment (MetS).

To date, diet-induced spontaneous and transgenic animal models have been widely used to study the effects of HFD and MetS in different organs and systems. Although these animal models can exhibit some of the components of different complex pathologies, mice and rats for example are deficient in cholesteryl ester transfer protein (CETP) and thus resistant to the development of heart disease, due to important differences in lipid metabolism with humans [19]. In addition, several rodent models do not develop important characteristics that are associated with cardiovascular disease such as hyperglycemia and atherosclerosis [20], as for larger canine and pig animal models [21], whose maintenance is necessarily very labor intensive and costly. On the other hand, rabbits, like humans, have abundant CETP in plasma, which acts as an important regulator of reverse cholesterol transport [22]. They are also very sensitive to dietary fat, so they can rapidly develop hyperlipidemia and cardiovascular lesions with morphological similarity to that of humans [23, 24], in addition to the similar hemodynamic and neurohumoral changes in obese humans [25–27]. Moreover, according to Bosze et al. [28], Russell and Proctor [29], and Dewre and Drion [30], a rabbit model is a valuable research model for cardiovascular human disease because rabbits are phylogenetically closer to humans than are rodents and they require easy handling and are small but large enough to allow nonlethal tracking of physiological changes.

For our study, and among animal models for biomedical and cardiovascular research, rabbit remains the most indicated because of their similarity with that of the human cardiac physiology [31, 32].

Only few studies on animal models focused on the influence of diets on the cardiovascular system, related to gender and age, but not on juvenile rabbits. For example, Hunter et al. [33] found that males and females fed with Western diet (high-fat high-sucrose diet) were affected differently.

So, the purpose of the present study is to investigate for the first time the influence of diet-induced overweight/obesity (high-fat diet (HFD)) on cardiac biochemistry and ultrastructure in both male and female prepubertal rabbits.

## 2. Material and Methods

### 2.1. Animals

Our study was carried out on 24 New Zealand prepubertal white rabbits weighting 1000 ± 50 g (12 male, 12 female). Animals were individually placed in stainless steel cages in an animal box (at room temperature 22–25°C, 50% relative air humidity, and 12 : 12 hour light : dark cycles).

Thereafter, sex-matched two-week-old rabbits were randomized regarding the type of food they received into 4 groups (for each, *n* = 6)—2 control groups, male (MC) and female (FC) which were fed a standard rabbit diet (100 g/day/rabbit) and 2 high-fat diet groups (MHFD, FHFD) which were fed with high-fat diet. All animal groups had free access to water.

### 2.2. Diet

The control groups were kept on a standard rabbit diet (rabbit chow bought at the national office of cattle feed, ONAB, Bejaïa, Algeria) consisting of 16.2% (*w*/*w*) crude protein, 3.0% (*w*/*w*) crude fat, 7.3% (*w*/*w*) crude ash, 14.8% (*w*/*w*) fiber, 12% (*w*/*w*) moisture, 2.1% (*w*/*w*) added mineral, 46% (*w*/*w*) carbohydrate with water, and 40 IU·kg^−1^ vitamin E. The HFD groups were kept on high-fat diet consisting of 100 g/day/rabbit of standard rabbit diet supplemented with 1 g of peanut butter + 0.5 g of sheep fat (diet supplemented with 1% of fat content).

The present study was approved by the Institutional Animal Care and Use Committee of the University of Sciences and Technology Houari Boumediene (Algiers, Algeria) and has been achieved according to the Executive Decree n°10–90 completing the Executive Decree n°04–82 of the Algerian Government, establishing the terms and modalities of animal welfare in animal facilities.

### 2.3. Experimental Protocol

After a week of acclimation to laboratory conditions, the rabbits had three months of diet, followed by monitoring. Body weight and plasma parameters were measured monthly and blood samples were withdrawn by puncture of the central ear vein. At the end of the experiment, the animals were fasted overnight and killed by decapitation for blood collection and tissue sampling.

#### 2.3.1. Blood and Tissue Harvesting

Blood was centrifuged and plasma stored at −40°C for biochemical analysis. The heart was rapidly excised and weighed; portions of the left ventricles were taken for histological and ultrastructural analysis, some were kept in Folch solution (1 V chloroform/2 V methanol) (Sigma-Aldrich®, Germany) for lipid determination [34], and others were homogenized in PBS (0.1 g of the left ventricle/0.9 mL PBS) and used for protein, calcium, and stress oxidant determinations.

#### 2.3.2. Biochemical Assays

Body and heart weights were compared between the groups (FC versus FHFD, MC versus MHFD, and FHFD versus MHFD). Lipid and glucose tests from blood and tissues were done using diagnostic enzyme assay kits (Spinreact®, Spain) such as total lipids (TL) [35], total cholesterol (TC) [36], cholesterol in high-density lipoprotein (HDL-C) [37], low-density lipoprotein (LDL-C) [38], triglycerides (TG) [39], phospholipids (PL) [34, 40], glucose (Glc) [41], CK-MB [42], calcium [43], and proteins [35].

Blood and tissue oxidant and antioxidant parameters were measured as follows: malonyldialdehyd (MDA) [44], ascorbic acid (AA) [45], antioxidant activity (AOA) [46], and uric acid [43].

#### 2.3.3. Thin-Layer Chromatography (TLC)

Neutral lipid thin-layer chromatography and phospholipid thin-layer chromatography were performed on the cardiac tissues of rabbits from different groups, in accordance with Prabha et al. [47] and Skipski et al. [48], respectively.

#### 2.3.4. Morphological and Histochemical Analysis

For standard histology, portions of the left ventricles were harvested, fixed in Bouin's aqueous solution, dehydrated in increasing concentrations of ethanol (70%, 95%, and 100%), cleared in toluene, and finally embedded in paraffin wax. Sections (3 *μ*m thick) were cut with a Leitz microtome and mounted on histological slides. After hydration, the sections were stained with Masson's trichrome and Sudan Black B as described by Martoja and Martoja [49] and Gabe [50]. Images were acquired using a light microscope (Carl Zeiss MicroImaging GmbH, Primo Star) fitted with a high-resolution camera (Canon PowerShot A640).

#### 2.3.5. Transmission Electron Microscopy

For electron microscopy studies, small fragments of the LV from rabbits of each group were minced and fixed immediately in cold 4% glutaraldehyde in 0.2 M phosphate buffer (pH 7.4) for 4 hours, followed by postfixation in 4% osmium tetroxide in 0.2 M phosphate buffer for 1 h. After dehydration in a series of graded alcohols, the fragments were embedded in epoxy resin and cut with an ultra Microtome LKB V. Semithin sections (600 nm) were stained with toluidine blue (TB).

Ultrathin sections (80 nm) were double stained with Uranyl-Less and lead citrate, in accordance with the studies of Benmeradi et al. [51] and Reynolds [52], respectively, and then examined under a Hitachi H7700 transmission electron microscope.

### 2.4. Statistical Analysis

All data were expressed as mean ± standard error of the mean (SEM). Comparisons were performed using a one-way variance analysis (ANOVA). *p* values lower than 0.05 (*p* < 0.05) were considered as indicative of a significant difference.

## 3. Results and Interpretations

### 3.1. Body Weight Evolution

**Figure 1:**
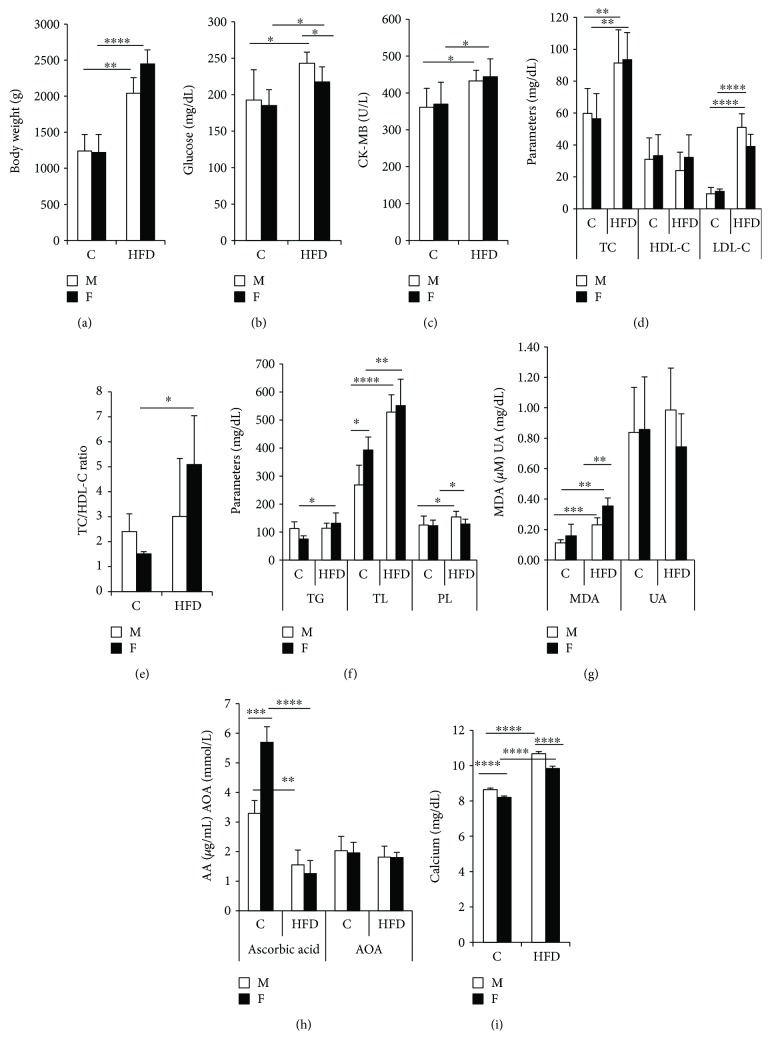
Body weight and plasma parameters in prepubertal rabbits. Body weight (a), glucose content (b), CK-MB content (c), cholesterol contents (d),TC/HDL-C ratio (e), lipid content (f), MDA and UA contents (g), AA and AOA contents (h), and calcium content (i). CK-MB: creatine kinase-MB; TG: triglycerides; TL: total lipids; PL: phospholipids; HDL-C: high-density lipoproteins; LDL-C: low-density lipoproteins; MDA: malonyldialdehyd; UA: uric acid; AA: ascorbic acid; AOA: antioxidant activity; C: control group; HFD: high-fat diet group; M: male; F: female. FHFD versus FC, MHFD versus MC, FHFD versus MHFD; mean ± SEM (^∗^*p* < 0.05, ^∗∗^*p* < 0.01, ^∗∗∗^*p* < 0.001, and ^∗∗∗∗^*p* < 0.0001).

The different results obtained in the two HFD groups are compared to their corresponding control groups. Under the high-fat diet group, we note a body weight increase in both the male (*p* < 0.01) and female groups (*p* < 0.0001) (Figure 1(a)).

### 3.2. Plasma Parameters

The female and male control parameters obtained before the start of the experiment are presented in Table 1. The HFD administered to prepubertal rabbits increases glycemia (Glc) (*p* < 0.05) and CK-MB activity (*p* < 0.05) in the male and female groups (Figures 1(b) and 1(c)). Total Lipids (TL) increase significantly in the male group (97%, *p* < 0.0001), while in the female group, the elevation reaches 40% (*p* < 0.01) (Figure 1(f)). We notice an elevation of triglyceride (TG) levels in the female group (76%, *p* < 0.05) (Figure 1(f)) and of phospholipid (PL) levels (23%, *p* < 0.05) in the male and female groups under the effect of HFD (Figure 1(f)). Total cholesterol (TC) increases similarly in the HFD male and female groups (*p* < 0.01) (Figure 1(d)), although LDL-C values appear to be higher for males (445%) than for females (260%), while HDL-C does not appear affected by this diet (Figure 1(d)).

There is a small rise of the TC/HDL-C ratio in the FHFD group (*p* < 0.05) (Figure 1(e)), and a significant increase of calcium levels in the HFD groups and among MHFD versus FHFD (*p* < 0.0001) (Figure 1(i)). The MDA increases in both the male (105%, *p* < 0,001) and female groups (124%, *p* < 0.01), with a high level among FHFD versus MHFD (*p* < 0.01) (Figure 1(g)). The uric acid (UA) and antioxidant activity (AOA) levels are similar between the male and female groups and do not appear affected by HFD (Figures 1(g) and 1(h)). Ascorbic acid (AA) decreases significantly in the male (*p* < 0.01) and female (*p* < 0.0001) groups with a high level among FC versus MC (*p* < 0.001) (Figure 1(h)).

### 3.3. Cardiac Parameters

#### 3.3.1. Cardiac Biochemistry

**Figure 2:**
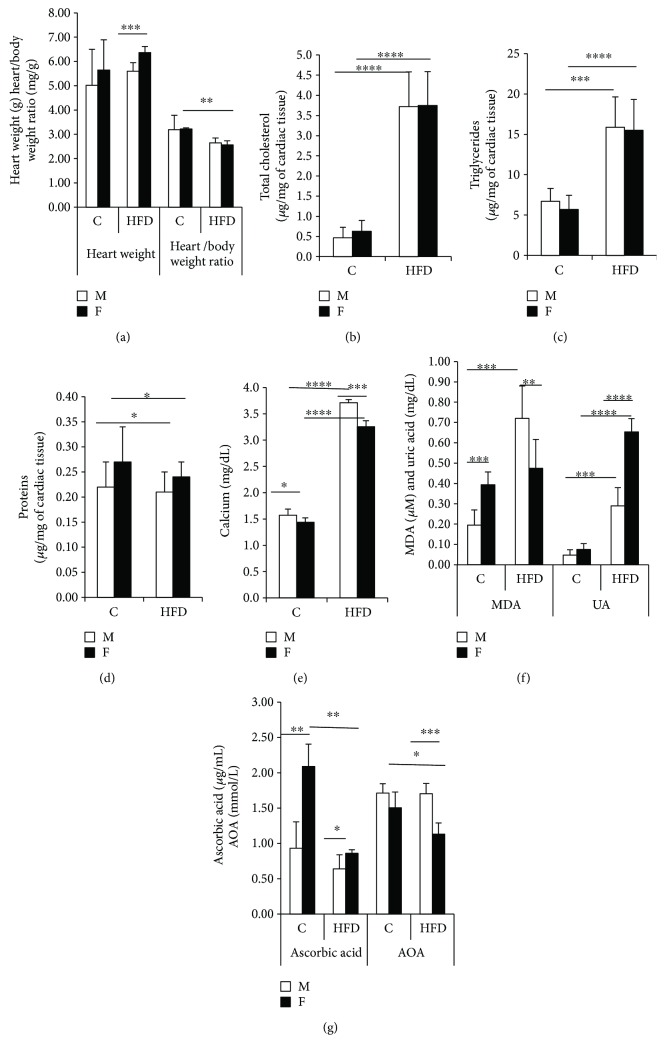
Heart weight and cardiac biochemical parameters in prepubertal rabbits. Heart weight and heart/body weight ratio (a), total cholesterol content (b), triglyceride content (c), protein content (d), calcium content (e), MDA and UA contents (f), and ascorbic acid and AOA contents (g). MDA: malonyldialdehyd; UA: uric acid; AOA: antioxidant activity; C: control group; HFD: high-fat diet group; M: male; F: female. FHFD versus FC, MHFD versus MC, FHFD versus MHFD; mean ± SEM (^∗^*p* < 0.05, ^∗∗^*p* < 0.01, ^∗∗∗^*p* < 0.001, and ^∗∗∗∗^*p* < 0.0001).

**Figure 3:**
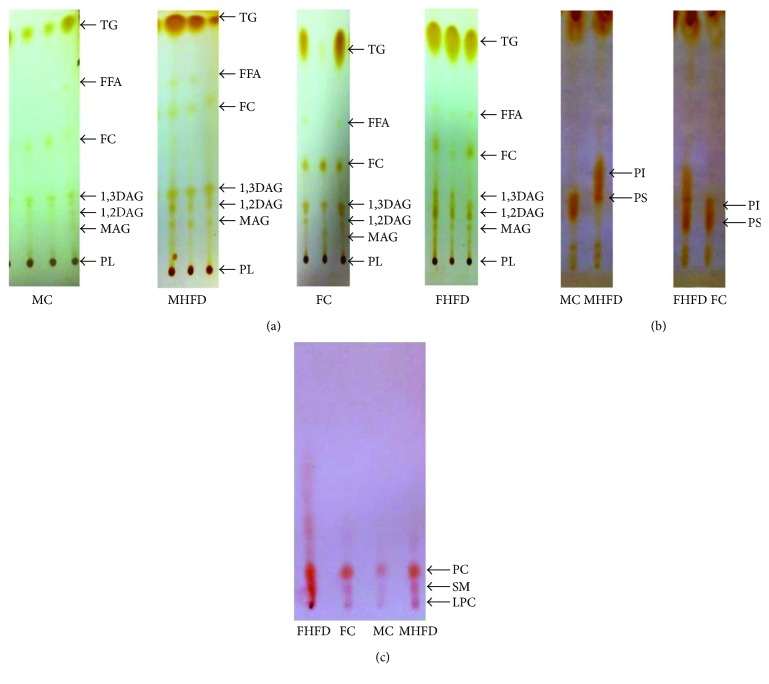
Prepubertal rabbit cardiac lipids: separation by thin-layer chromatography. Neutral lipid detection by iodine vaporization (a). PL: phospholipids; MAG: monoacylglycerol; 1,2DAG: 1,2 diacylglycerol; 1,3DAG: 1,3 diacylglycerol; FC: free cholesterol; FFA: free fatty acid; TG: triacylglycerol. Phospholipid detection by iodine vaporization (b). PI: phosphatidylinositol; PS: phosphatidylserine. Phospholipid detection by ninhydrine (c). PC: phosphatidylcholine; SM: sphingomyelin; LPC: lysophosphatidylcholine; FC: female control group; MC: male control group, FHFD: female high-fat diet group; MHFD: male high-fat diet group.

Our study shows a nonsignificant elevation of heart weight in prepubertal MHFD, as well as in FHFD groups, but a great difference is noted between FHFD and MHFD (*p* < 0.001) (Figure 2(a)). The heart/body weights ratio shows a smaller value in FHFD versus FC indicating that the evolution of the body exceeds that of the heart in females (Figure 2(a)). There is a very significant increase of TC in both the female and male (*p* < 0.0001) HFD groups (Figure 2(b)). Also, TG raises significantly in both the male (*p* < 0.001) and female HFD groups (*p* < 0.0001) (Figure 2(c)). The results obtained by biochemical assays are consistent with those obtained by TLC for neutral lipids (Figure 3(a)) and PL (Figures 3(b) and 3(c)). There is a small raise of proteins levels in the HFD groups (*p* < 0.05) (Figure 2(d)). A significant increase of calcium levels in the HFD groups compared to their control counterparts is found (*p* < 0.0001), and also between MHFD versus FHFD (*p* < 0.001) (Figure 2(e)). We notice a significant raise of MDA in the MHFD compared to MC (*p* < 0.001) and to FHFD (*p* < 0.01), also between FC versus MC (*p* < 0.001) (Figure 2(f)). The UA increases significantly in the HFD male and female groups (*p* < 0.001 and *p* < 0.0001, resp.) and also among FHFD versus MHFD (*p* < 0.0001) (Figure 2(f)). The level of AA is high among FC versus MC (*p*<0.01); under HFD effect, it decreases in the female group (*p* < 0.01) (Figure 2(g)). The antioxidant activity (AOA) decreases only in FHFD (*p* < 0.05), resulting in a significant difference among MHFD versus FHFD (*p* < 0.001) (Figure 2(g)).

#### 3.3.2. Cardiac Morphology and Ultrastructure

**Figure 4:**
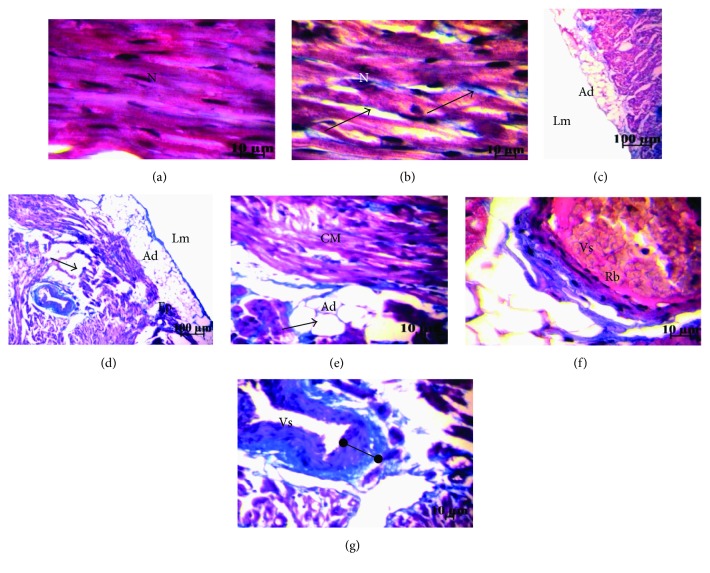
Histology of left ventricular heart of prepubertal female rabbits. Masson's trichrome staining. Normal diet group (a, c, f) and high-fat diet group (b, d, e, g). Under the high-fat diet group, small collagen deposits stained in blue are noted (b, arrow); spaces between cells are apparent (b, bold arrow) with a cardiomyocyte disorganization (b, e); important adipose tissue deposits appears under the epicardium, the surrounding vessel, and also between cardiomyocytes (d, e); and the vascular wall is more thick with disoriented nuclei. Ad: adipocyte; CM: cardiomyocyte; Lm: lumen; N: nucleus; Rb: red blood corpuscle; Vs: vessel.

**Figure 5:**
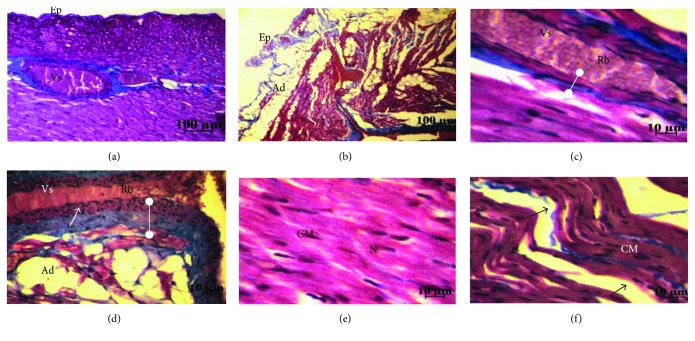
Histology of the left ventricular heart of prepubertal male rabbits. Masson's trichrome staining. Normal diet group (a, c, e) and high-fat diet group (b, d, f). Under the high-fat diet group, the vascular wall is more thick with disorganized nuclei (d, arrow); adipose tissue appears under the pericardium, surrounding the vessel, and between cardiomyocytes (b, d); spaces between cells are apparent with cardiomyocyte disorganization (f, arrows). Ad: adipocyte; CM: cardiomyocyte; Ep: epicardium; N: nucleus; Rb: red blood corpuscule; Vs: vessel.

**Figure 6:**
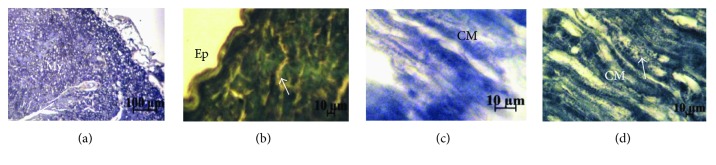
Histochemistry of the left ventricular heart of prepubertal female and male rabbits. Sudan Black B staining. Female normal-diet group (a), female high-fat diet group (b), male normal-diet group (c), and male high-fat diet group (d). Positive Sudan Black B staining of lipids in high-fat diet female and male groups with characteristic deposition of black granules in the CM stained with black (b, d, arrows). CM: cardiomyocyte; My: myocardium.

**Figure 7:**
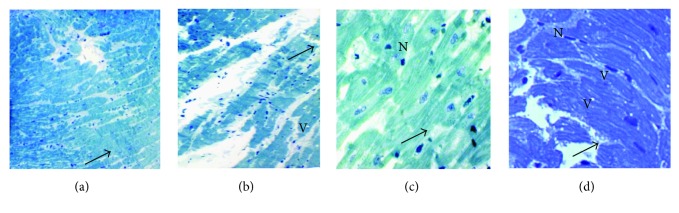
Photomicrographs of semithin sections in the cardiac tissue of prepubertal female and male rabbits. Toluidine blue staining 100x (a, b) and 400x (c, d). Female normal-diet group (a), female high-fat diet group (b), male normal diet group (c), and male high-fat diet group (d). The cells adhere to each other in the female and male normal-diet groups (a, c, arrows) comparing to the high-fat diet groups; so under HFD effect, the cells are less contiguous. An area of focal degeneration is seen in the 2 high-fat diet groups (b, d, arrows) especially in FHFD with multiple vacuolization (V) present between the cardiomyocytes. N: nucleus; V: lipid vacuoles.

The Masson's trichrome staining shows in the heart of FC and MC groups a homogeneous tissue of joined cardiomyocytes, a regular structure of the three layers of cardiac vessels (Figure 4), whereas in FHFD, we find collagen deposits stained in blue, spaces between cells, cardiomyocytes disorganization, adipose tissue deposits under the pericardium, and surround vessels and between cardiomyocytes and a thicker vascular wall with reoriented nuclei (Figure 4). The same observations were obtained in MHFD with less intensity, especially regarding adipose and collagen deposits (Figure 5). The Sudan Black B staining characterizes the lipid deposits, which are marked in both HFD groups (Figure 6). We observed less contiguous cells and more important areas of focal degeneration in FHFD compared to MHFD, with multiple vacuolization present between the cardiomyocytes and striations (semithin sections) (Figure 7).

**Figure 8:**
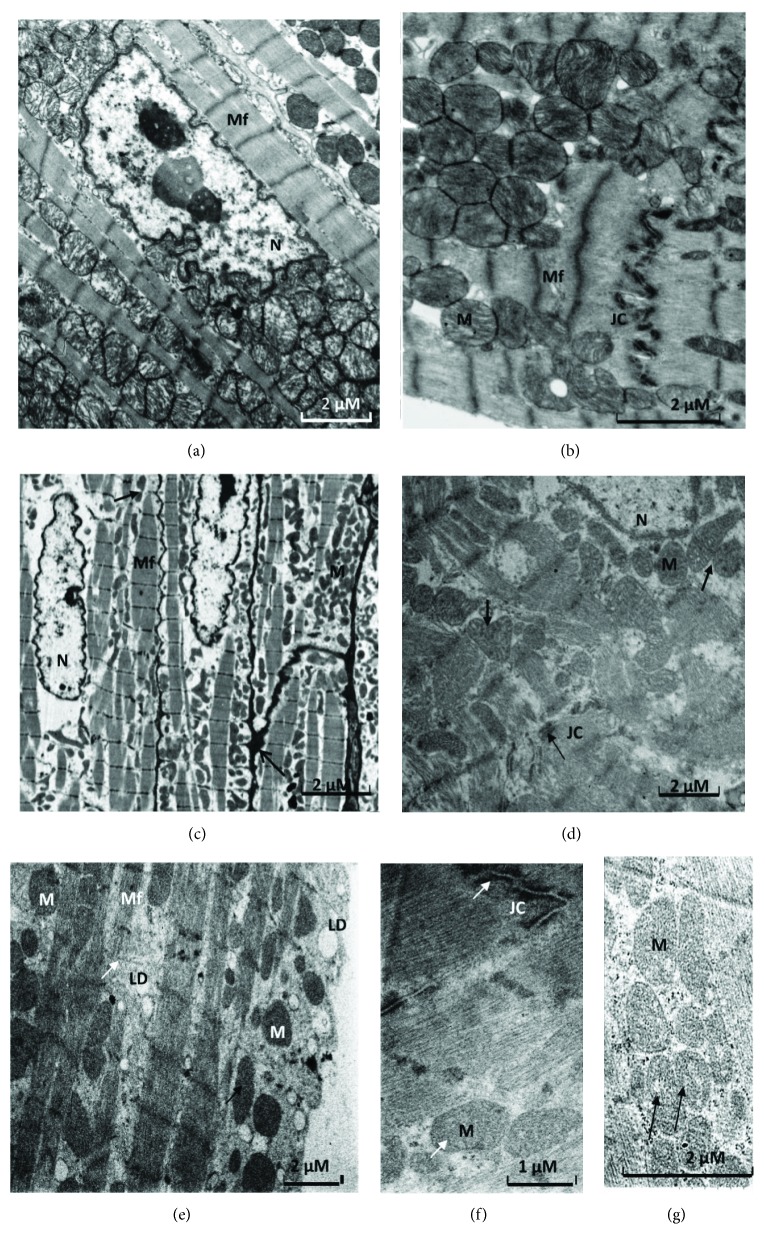
Electron photomicrographs of ultrathin sections in the cardiac tissue of prepubertal female rabbits. Normal-diet group (a, b) and high-fat diet group (c, d, e, f, g). Under the high-fat diet group, there is a decrease of myofibrils (Mf) which appear degenerated with sarcomere disorganization (e), enlargement of intercellular junctions (c, arrow) and abundant sparse mitochondria (M) (c, e, bold arrows), damaged junctional complexes (JC), and multiple lipid droplets (LD) (d, e, f, arrows). Damaged mitochondria appear swollen, elongated with variation in their size, disorganization, and loss of their cristae, with some figures evoking mitochondria duplication and others presenting electron-dense material in the matrix (d, e, f, g, arrows). JC: junctional complex; M: mitochondria; Mf: myofibrils; N: nucleus; LD: lipid droplet.

**Figure 9:**
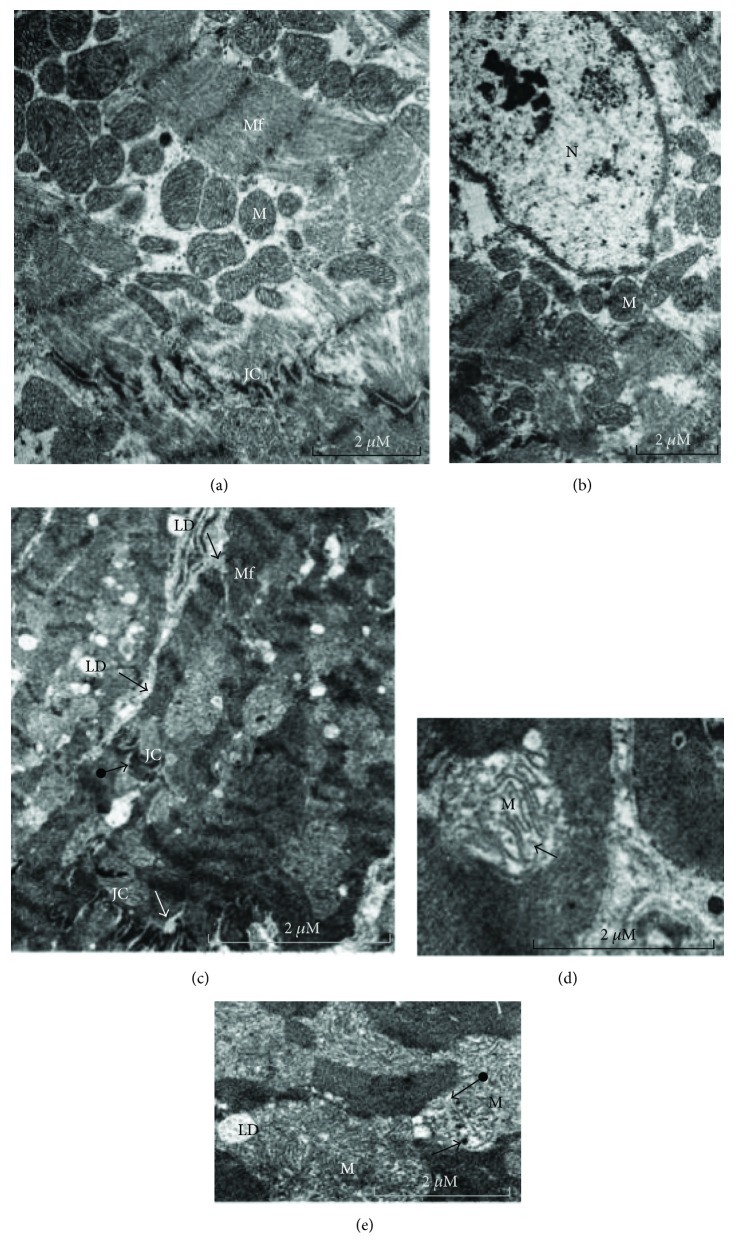
Electron photomicrographs of ultrathin sections in the cardiac tissue of prepubertal male rabbits. Normal diet group (a, b) and high-fat diet group (c, d, e). Under the high-fat diet group, mitochondrial ultrastructure (M) showed evidence of morphological abnormalities with dense material deposits (e, bold arrow), pronounced variation in their size, disorganization of cristae which appear significantly deformed, and mitochondria duplication (bold arrows) (d, e). The junctional complexes (JC) are enlarged (c). There is a decrease of myofibrils (Mf) which appear degenerated with sarcomere disorganization (c, arrow), multiple lipid droplets (LD) (c, e). JC: junctional complex; M: mitochondria; Mf: myofibrils; N: nucleus; LD: lipid droplets.

Under high-fat diet, ultrastructural analysis shows a decrease of myofibrils which appear degenerated with sarcomere disorganization, enlargement of intercellular junctions, sparse mitochondria, damaged junctional complexes, and multiple vacuolization. Damaged mitochondria appear swollen, elongated with pronounced variation in their size, disorganization of their cristae, with some figures evoking mitochondria duplication, and others presenting electron-dense material in the matrix, localized essentially in the perinuclear space and between the myofilaments (Figure 8). The ultrastructure of the MHFD heart is similar to that of FHFD (Figure 9), but presents bigger mitochondria.

## 4. Discussion and Conclusion

The aim of this study is to analyze the gender-specific effect of early high-fat diet feeding on cardiac biochemistry and ultrastructure of prepubertal rabbit, an animal model which mimics the main changes that occur in humans.

High-fat diet induces an elevation of body weight in both male and female prepubertal rabbits; these results corroborate those obtained in other studies with analogous diets on adult female [25] and male rabbits [26, 53–55]. The weight excess observed in our prepubertal models may be the cause of pathologies declared in adulthood, similarly to humans. Our study does not show a significant variation in body weight related to the gender. Although Strong and Kelder [15] and Tolfrey et al. [56] reported a relationship between these two parameters attributed to gonadal hormone influence, the molecular mechanisms involved remain not understood.

According to the study of Arias-Mutis et al. [57], the main components of rabbit MetS is similar to those of human, by the administration of high-fat and high-sucrose diet that induced alterations in the glycemia and the lipid profile revealed by an increase in fasting glucose, TG, and LDL, which is similar to those of our results. Feeding our male and female rabbits with a HFD during 12 weeks induces high TG, PL, TC, and LDL-C levels; high visceral fat deposits; high fasting glucose plasma levels, and high TC/HDL-C ratio. The same results are obtained by Zhang et al. [58] on adult male and female rabbits fed with HFD during 20 days and by Maneschi et al. [59] on male rabbits, indicating the link between the diet and cardiovascular risk.

As mentioned by Alarcón et al. [60], the symptoms cited below are also part of metabolic syndrome. For Hunter et al. [33], metabolic syndrome (MetS) is associated with enhanced oxidative stress. Alarcón et al. [60] mentioned a cardiometabolic risk with such parameter variations.

Jain [61] found a relationship between glucose concentration and oxidative stress in red blood cells. Hyperglycemia can generate free radicals by autooxidation and glycation of proteins [62].

In our control rabbits, protection via ascorbic acid in females is better compared to males; however under HFD, the AA levels decrease in the two genders as mentioned in the study of Moor de Burgos et al. [6], Sethi et al. [63], and Sabitha et al. [64].

The HFD administered to our rabbits enhances lipid oxidation. This is indicated by MDA rise according to Yu et al. [55], Zhang et al. [65], Sethi et al. [63], Apaijai et al. [66], and Pipatpiboon et al. [67]. It also minimizes the total antioxidant protection by AOA and ascorbic acid level reduction as in the study of Moor de Burgos et al. [6]. According to Adamopoulos et al. [68], women tend to have lower uric acid level than men, probably because of the uricosuric effect of estrogens. However, in our study on prepubertal rabbits, we find no difference in plasma UA levels between FHFD and MHFD, but there was a significant elevation of UA in FHFD compared to their control counterpart and to that in MHFD. Since the protein level has not changed, it can be assumed that UA would have a different origin such as xanthine oxidoreductase, an enzyme expressed in adipocytes with critical function in the adipogenesis process [69].

Some studies have noted that an elevated level of uric acid predicts the development of obesity [70] and induces cardiovascular diseases by inhibition of the endothelium function [71]. Another study indicated uric acid as a prooxidant, either by generating radicals during its degradation or by stimulating NADPH oxidase [72]. In female prepubertal rabbits, we note a decrease in heart/body weight ratio. This result, also obtained by Wang et al. [73], indicates a body growth greater than cardiac growth and is visualized by massive visceral fat accumulation.

According to the studies on adult female and male rabbits of Waqar et al. [22] and Zhang et al. [58], after consuming high-fat diet, a cardiovascular adipose accumulation is observed in the histological sections. So, we notice adipose tissue deposits (TG reserve) between cardiomyocytes and in the epicardium, especially in FHFD (Masson's trichrome staining), according to Wang et al. [74] and Eppel et al. [75]. In our HFD groups, very small collagen deposits are observed, notably in FHFD (Masson's staining). According to Lucas et al. [76], it seems that the heart weight of our prepubertal models is not affected with such intensity by HFD, given their age and the relative duration of the diet (12 weeks).

According to many studies on the subject, HFD induced aortic injuries in rabbits, which can lead with time to the development of atherosclerosis.

Our work on thoracic aortas of prepubertal rabbits fed with HFD, compared to their control counterparts, showed high levels of lipid deposits, agglutination of blood elements to the intima, and disorganization of smooth muscle cells in the 2 genders with an alteration of the elastic fibers especially in MHFD aortas (unpublished results).

In our study, high-fat diet seems to raise the cardiac TL, especially the PL rates (PC, SM, LPC, and PS) in both male and female (estimated by TLC and Sudan Black staining). We also notice that cardiac TG and TC are higher in FHFD than in MHFD. Moreover, as these parameters are the most incriminated in cardiovascular diseases, by hydrolysis-reesterification cycles [77–79], the females are more exposed to pathologies. So, under HFD, our study shows a pronounced cardiac alteration in females compared to males that can be explained by the absence of the protective female hormone, since our models are still immature. This observation is in agreement with that of Bell et al. [80, 81] on young adult rodents at prime estrogenic life stage, indicating the absence of a female protected state in the context of hypertrophy.

These cardiac lipid disorders lead to unbalanced oxidative status, according to the results of Aragno et al. [53] on adult male rabbits fed with HFD for 12 weeks. Nevertheless, we observe an imbalance in oxidative status accentuated in female fed with HFD, with a rise in UA, and diminutions in AA and AOA.

For Dirkx et al. [82] and Goldberg et al. [83], the produced ROS can lead to mitochondrial dysfunction and cardiac hypertrophy. Oxidative stress may be important in the development of coronary artery disease, the heart being poorly protected against oxidative stress [12]. It is established that abnormal lipid metabolism is associated with impaired mitochondrial structure and function [84], cardiac efficiency loss [85], and cardiomyopathy particularly due to lipid-induced apoptosis [86]. Bell et al. [87] data show that basal cardiomyocyte contractile performance in nonhypertrophic cardiomyocytes is lower in females versus males, but this difference is lost when myocytes present hypertrophy.

Areas of focal degeneration in cardiac tissue are noted primarily in the FHFD group, with multiple vacuolization present between the cardiomyocytes, as seen in semithin slices. Ultrastructural analysis of prepubertal FHFD and MHFD cardiac tissues shows modifications mentioned by many authors in adult models. Our results obtained in both male and female models fed with HFD show an increase in septal thickness with a large accumulation of lipids. We note an elevation of the total mitochondria area and sparse mitochondria, which express a lesion of the mitochondrial system according to Sycz [88] on rabbits fed with HFD that can be explained by the large energy need of the heart, with evidence of morphological abnormalities. We register mitochondrial swelling and elongation in both MHFD and FHFD cardiomyocytes, with the presence of a dense material deposit identified by Kloner et al. [89] and Solenski et al. [90], as calcium overloading, an important marker of mitochondrial damage. We observed disorganization of myofibrils and cristae with some figures of mitochondria duplication/separation and loss of sarcomere integrity. Rózycka et al. [91] reported similar observations on rabbits fed with HFD and Shirakawa et al. [92] on immature rabbit hearts after infusion/reperfusion.

In addition, the enlargement of intercellular junctions, damaged junctional complexes (JC), and the multiple vacuolization that we observed in HFD groups are mentioned by Dong et al. [93]. We also note a thickening of striated areas especially in FHFD.

In the MHFD left ventricle, we observe larger mitochondria than in the FHFD left ventricle with disorganized separated cristae, accordingly to the data reported by Bostick et al. [94] on mice fed with Western diet. According to Halestrap and Pasdois [95] and Ong and Hausenloy [96], larger mitochondria are implicated in better protection against I/R, possibly by being able to accommodate more Ca^2+^ before the mitochondrial permeability transition pore opens.

In our study, we find a significant difference in heart calcium rate between MHFD and FHFD. Males fed with HFD have higher rates. The large mitochondria that we observed in MHFD indicate an important lipid catabolism process, such as betaoxidation, which affects fatty acids (diet FFA and TG), since the energy demand is high. The combination of increased calcium content and energy production results in a stimulation of cardiac activity, which appears to be more important in MHFD than in FHFD. According to Stanley et al. [97], the uptake of FAs by the heart through FA oxidation may lead to excessive storage of FAs as TG in cardiac myocytes as what we have observed in the heart of FHFD. Bell et al. [87] indicate that the *β*-adrenergic response is less pronounced in healthy cardiac tissues/myocytes of females versus males. This difference is more likely attributable to downstream signaling intermediates as PKA or cAMP [98]. Recent studies [99, 100] suggest that local myocardial androgen-to-estrogen conversion determines sex specificity in hypertrophy. Under stress conditions (as HFD), the level of local steroid may become important in modulating function.

Disturbance of myocardial lipid metabolism may induce stress in sarcoplasmic reticula, Ca^++^ transport perturbation (as we obtain in MHFD), resulting in myocardial dysfunction and heart failure [101]. Different pathways are involved in myocardial function alterations, such as PKA, caMK2D [102], and PIK fyve, via mitochondrial damage [103].

Our results are in agreement with those obtained in adult models of many authors, mentioning that diets with high fat content conduct to a number of metabolic perturbations including dyslipidemia by altering the proportion of VLDL/LDL and HDL, causing oxidative stress and mitochondrial dysfunction [104].

To empower our results, we intend to complete our study of the HFD effects on prepubertal female and male rabbits by performing electrocardiographic analysis of cardiac function and remodeling, also by measuring systolic and diastolic pressures in the two rabbit genders.

## 5. Conclusion

In summary, this experimental investigation demonstrates that high-fat feeding induces plasma lipid disorders and CK-MB increase in the two prepubertal genders. Male plasma parameters are more affected by this HFD than those of females. On the other hand, body weight, heart/body weight ratio, heart TG, and UA levels are significantly increased in FHFD when the protective parameters decrease (AA, AOA). The Ca^++^ transport disturbance (contraction/relaxation process alteration) result in cardiac pathophenotypes and structural abnormalities (mitochondrial enlargement, duplication, and cristae loss). This is an interesting issue in view of the association between overweight, dyslipidemia, oxidative stress, and heart diseases in prepubertal models. The lipotoxicity we obtained by this HFD via ROS overproduction and mitochondrial damage may result in activation of apoptotic cascades and play roles in the pathogenesis of cardiomyopathy in both female and male, even so at the prepubertal stage.

Our results demonstrate the gender-specific effect of high-fat feeding on cardiac ultrastructure in prepubertal rabbits. Many authors consider that females have better cardioprotection, but only a few circulating parameters are in favor of this hypothesis. On the other hand, female cardiac tissue is more affected by the HFD than the left ventricle of male. According to our findings, studies mentioned that young women are at greater risk of developing heart failure in conditions of overnutrition/obesity. We agree with some authors who studied young adult animal models at prime estrogenic life stage and concluded that the conventional view of female estrogen cardioprotection may require further investigations. It is expected that such data may be useful for the implementation of preventive strategies during childhood, including the control of overweight, irregular glycemia, prooxidant levels, and dyslipidemia, which could help early prevention of cardiovascular diseases.

## Figures and Tables

**Table 1:** Initial body weight and plasma biochemical parameters of prepubertal female and male rabbits.

Parameters	Male	Female
Body weight (g)	1011 ± 172.27	945 ± 124.67
Glc (mg/dL)	121.62 ± 3.65	122.86 ± 2.5
TG (mg/dL)	85.58 ± 28.39	74.29 ± 17.05
TL (mg/dL)	264.18 ± 56.18	212.31 ± 81.70
PL (mg/dL)	110.38 ± 28.74	120.19 ± 15.12
TC (mg/dL)	64.7 ± 13.94	52.77 ± 9.5
HDL-C (mg/dL)	23.40 ± 4.43	21.06 ± 6.27
LDL-C (mg/dL)	12.20 ± 3.34	8.98 ± 6.48
UA (mg/dL)	0.92 ± 0.31	0.94 ± 0.56
TBARS (*μ*M)	0.08 ± 0.02	0.1 ± 0.04
AA (*μ*g/mL)	3.85 ± 1.43	2.61 ± 0.97
AOA (mmol/L)	1.98 ± 0.19	1.82 ± 0.05
CK-MB (U/L)	343.09 ± 31.73	330.43 ± 26.48
Calcium (mg/dL)	7.84 ± 0.07	7.66 ± 0.04
